# Towards institutionalizing HTA in Ethiopia: using a political economy analysis to explore stakeholder perspectives and assessing capacity needs

**DOI:** 10.1017/S0266462325000170

**Published:** 2025-04-03

**Authors:** Daniel Asfaw Erku, Ararso Desalegn, Tesfaye Mesele Mekonnen, Ermias Dessie, Firmaye Bogale Wolde, Sabit Ababor Ababulgu, Paul A. Scuffham, Damian Walker, Rabia Sucu, Samuel Abera

**Affiliations:** 1 Health Economics and Financing, Global Health Systems Innovation, Management Sciences for Health, Arlington, VA, USA; 2 Centre for Applied Health Economics, School of Medicine, Griffith University, Brisbane, QLD, Australia; 3 Economic, Policy and Innovation Centre for Health Systems (EPIC Health Systems), Addis Ababa, Ethiopia; 4 Technology Transfer and Research Translation Directorate, Ethiopian Public Health Institute, Addis Ababa, Ethiopia; 5 Healthcare Financing and Economics Technical Advisor, Strategic Affairs Directorate, Ministry of Health, Addis Ababa, Ethiopia; 6 Knowledge Translation Center for Health, Ethiopian Public Health Institute, Addis Ababa, Ethiopia

**Keywords:** health technology assessment, Sub Saharan Africa, decision-making, capacity building, policy making, priority setting

## Abstract

**Background:**

As Ethiopia advances towards efficient resource utilization and UHC through strategic health purchasing, the institutionalization of HTA will play a critical role. This study aims to identify key stakeholders, analyze the political economy surrounding HTA and priority setting in Ethiopia, and assess existing skills and capacities for a robust and sustainable HTA system.

**Methods:**

We employed a mixed-method approach, combining 16 key informant interviews, 24 document reviews, and a cross-sectional survey (n=65) to assess national HTA capacity. We employed the Walt and Gilson policy analysis triangle framework, alongside Campos and Reich’s framework, to evaluate the context, process, content, and actors influencing HTA institutionalization, and to explore the complex interplay of institutions, positions, power, and interests among various stakeholders.

**Results:**

While there is a general commitment to implementing HTA across various government agencies and stakeholder groups, the institutionalization process faces several challenges, involving multiple agencies with overlapping mandates, raises bureaucratic challenges and potential conflicts, risking horizontal fragmentation as agencies compete for authority, budget, and influence. The involvement of other key stakeholders, such as professional associations, patients, and the public, is notably lacking. Challenges such as limited HTA expertise, high professional turnover, and gaps in specific HTA knowledge areas persist, with capacity-building efforts often failing to address organizational needs effectively.

**Conclusions:**

The complexity of HTA institutionalization in Ethiopia underscores the necessity of managing intricate inter-agency dynamics, establishing a robust legal framework for an inclusive and transparent HTA process, building local capacity, and securing sustainable, domestically aligned funding.

## Background

The core of universal health coverage (UHC) lies in providing healthcare services that are not only efficient and of high quality but also accessible to everyone, irrespective of their socio-economic background, while also ensuring financial risk protection (1). Achieving UHC requires generating resources and directing them toward priority health needs to maximize impact (2;3). In low- and middle-income countries (LMICs) like Ethiopia, where resources are limited and reliance on out-of-pocket payments and donor funding is high, systematic prioritization and strategic allocation of public health budgets are vital (4–7). Central to this effort is the development of health benefit packages—services funded through pooled resources—and the establishment of robust institutions and processes to guide these decisions (3;8–11).

Health Technology Assessment (HTA) offers a structured approach to prioritize health interventions by evaluating their clinical effectiveness, safety, cost-effectiveness, and social implications (12). HTA informs decisions on adopting, reimbursing, or covering health interventions, ensuring they align with population needs and financial constraints. It also estimates budget impacts and identifies barriers that could exacerbate inequities. The WHO has emphasized HTA’s role in UHC and adopted Resolution WHA67.23 (“Health intervention and technology assessment in support of universal health coverage”) at the Sixty-seventh World Health Assembly to promote its integration into health systems (7).

Ethiopia, with its population surpassing 120 million, is adopting this approach to optimize the use of its limited health sector resources in its pursuit of UHC. A key element of this strategy is the establishment of an HTA unit, and the Ministry of Health has developed a national HTA roadmap to institutionalize explicit priority-setting mechanisms based on an extensive situation analysis (13). However, institutionalizing HTA requires balancing diverse stakeholder interests and navigating the complex political economy influencing its integration (14;15). Sustaining HTA also depends on building the capacity to generate and use HTA outputs, which involves assessing individual skills, organizational resources, and regulatory frameworks (16–19). Despite its importance, Ethiopia has yet to systematically evaluate its political economy and capacity for HTA. This study aims to (i) identify key stakeholders and analyze the political economy shaping HTA institutionalization, and (ii) assess the skills and capacities needed for a robust, sustainable HTA system.

## Methods

### Study design and conceptual framework

We employed a mixed-method approach, combining key informant interviews, document review, and a quantitative assessment of HTA capacity to examine the HTA landscape and priority setting in Ethiopia. The Walt and Gilson policy analysis triangle framework (20;21) was used to analyze the context, process, content, and actors influencing HTA institutionalization. To complement this, Campos and Reich’s framework (15;22–24) was applied to explore the complex interplay of institutions, positions, power, and interests among stakeholders, highlighting how these factors interact and compete to influence policymaking. This approach allowed us to uncover patterns of collaboration, conflict, and cooperation among stakeholders, offering a nuanced understanding of HTA implementation dynamics. The study examined Ethiopia’s current and historical approaches to priority setting, alignment with UHC, and other national strategies. Key stakeholders were mapped, and their roles and power dynamics analyzed, conceptualizing power as dispositional (money, knowledge, reputation), relational (influence), and organizational (rules, bargaining) (25). Institutions, both formal (governing structures, agreements) and informal (unarticulated rules), were assessed to understand their role in shaping priority-setting processes. Ethical approval was obtained from the University of Gondar (Ref: VP/RTT/05/250/2023), and participants provided informed consent.

### Policy document review

A national HTA Technical Working Group (TWG) was established in 2023 through a collaborative effort by the Ministry of Health and the Ethiopian Public Health Institute, along with key stakeholders such as the Ethiopian Health Insurance Services, Ethiopian Pharmaceuticals Supply Services, and the Ethiopian Food and Drug Administration, and supporting members from academia and professional associations. The TWG was tasked with developing a roadmap to strengthen HTA processes in Ethiopia. As part of this effort, the TWG gathered and reviewed a diverse portfolio of policy documents (n = 24), including legislative and policy documents, and held a series of consultative meetings. For this study, these documents and minutes from a series of TWG meetings were analyzed to provide insights into the status and role of HTA in Ethiopia’s health system, as well as the priority-setting practices employed across different government agencies and work environments.

### Key informant interview

After reviewing relevant documents, we conducted an extensive stakeholder mapping process in collaboration with the TWG, guided by a structured stakeholder mapping tool (26). Key informants were identified using a snowballing technique and input from the TWG. We then applied purposive sampling to ensure broad representation from organizations, groups, and individuals critical to HTA and priority-setting processes. Invitations were sent via email, letters, and phone calls. Between December 2022 and July 2023, we conducted 16 interviews with stakeholders, including representatives from government agencies (e.g., Pharmaceutical Supply Agency, Health Insurance Service), academic institutions, NGOs, professional associations, the private sector, and patient groups. Interviews lasted 30–60 minutes and were conducted in English via Zoom by two authors (DE and SZ). All interviews were audio recorded for transcription and analysis. The first section of the interview guide focused on contextual factors such as historical decision-making practices, ideologies, values, and the framing of HTA and priority setting. It examined how these elements influence the design, adoption, and implementation of priority-setting systems. The second section centered on actors, their roles in decision-making, and capacity-building needs. It explored the institutionalization of HTA in Ethiopia, its effects on resource distribution, and its broader impact on health financing and equity reforms. Details of the broader themes and the topic guide tailored for each stakeholder group are available in the supplementary file.

### Quantitative capacity assessment survey

We sent an online HTA capacity assessment survey via email and social media platforms to participants identified through purposive sampling, which involved individuals working in research institutes, universities, non-governmental organizations, health insurance agencies, and the Ministry of Health. The survey was open for a period of two months, and we sent two reminder emails during this time. The survey was designed based on an extensive review of literature on HTA and priority setting in LMICs, and it incorporated elements from an existing capacity assessment tool (27). The survey consisted of four sections: (i) sociodemographic characteristics of the respondents, (ii) familiarity with key HTA concepts and methods, (iii) priority areas and health technologies for HTA implementation in Ethiopia, and (iv) participant confidence in HTA skills, and identified barriers to HTA production and use in Ethiopia. The survey utilized a five-point Likert scale, for example, ranging from “Not at all or slightly familiar” to “Very familiar,” to assess participants’ familiarity with various aspects of HTA. The survey consisted of questions related to the purpose, scope, and application of HTA, as well as knowledge of literature searching methods, clinical study designs, economic evaluations, and equity concepts.

### Data analysis

All included documents and interviews were coded using NVivo V.12 software. To ensure consistency, an initial codebook was developed based on the research questions. Two authors piloted this codebook by conducting initial analyses on a sample set of data, which included two policy documents and one interview. During this process, any coding discrepancies were discussed and resolved collaboratively. After finalizing the codebook, one researcher (DE) proceeded to code the remaining interviews and documents, ensuring uniformity and coherence throughout the analysis. Quantitative survey data were analyzed using SPSS version 28, employing descriptive statistics such as means, frequencies, and percentages, with results presented in tables. Findings from policy documents, qualitative interviews, and the survey were analyzed separately and synthesized to provide a comprehensive view of HTA in Ethiopia, identifying challenges and key areas for improvement toward a sustainable HTA system.

## Results

Sixteen key informants were interviewed, representing stakeholders from key health agencies, academia, patient groups, and the private sector. Additionally, 65 participants completed the HTA capacity assessment survey. Most survey respondents (84.6 percent) were from research institutes or universities, followed by the Ministry of Health or affiliated agencies (9.3 percent) and NGOs (6.1 percent). Sociodemographic details are in Table 1.

**Table 1.** Socio-demographic characteristics of interview participants and survey respondents

Variable	Survey respondents (N = 65)	Interview participants (n = 16)
	Frequency (%)	Frequency (%)
Gender		
Female	5 (7.7)	4 (25)
Male	60 (92.3)	12 (75)
Work affiliation		
Research institute or university	55 (84.6)	3 (18.8)
Office within the Ministry of Health^a^	6 (9.3)	11 (0.67)
Non-Governmental Organization	4 (6.1)	2 (12.5)
Highest level of education		
Bachelor	1 (1.5)	
Master	41 (63.1)	
Doctoral	23 (35.4)	

a Includes the ministry of health or other federal agencies within in the ministry.

### Healthcare priority-setting approaches in Ethiopia

The Ethiopian health system’s reliance on a mix of government contributions, external funding, and out-of-pocket expenditures, combined with its decentralized approach to healthcare, shapes the context in which healthcare priority setting is conducted and reimbursement decisions are made. Priority setting for healthcare occurs at different governmental levels, from national to regional levels. Over recent decades, Ethiopia has emphasized decentralizing healthcare services, focusing on health promotion, disease prevention, and essential curative services. This strategic direction is encapsulated in key government documents like the Essential Health Service Package (EHSP) (28), Pharmaceutical Procurement List (29), and Health Insurance Benefit Package, which collectively guide service delivery and reimbursement decisions. In developing and revising the EHSP, the government adopted a multi-criteria decision analysis (MCDA) approach, ensuring the process was participatory, inclusive, and evidence-informed, following a clear roadmap (30). The evidence-informed prioritization in the EHSP revision indicates steps towards an explicit, evidence-informed healthcare priority-setting approach by the government. Yet, without a consistent stream of domestic funding and local technical expertise to generate and interpret data relevant to Ethiopia’s context for HTA, the sustainability of such priority-setting activities remains uncertain. In addition, the extent to which other policy documents have been developed and/or revised using HTA, MCDA, or similar explicit priority-setting approaches remains unclear. For instance, although the Pharmaceuticals Procurement List (PPL) has been developed by a dedicated taskforce that critically reviews previous pharmaceutical lists and trends in pharmaceutical requests, and adopted international criteria from organizations like WHO to develop contextualized criteria, the explicit application of HTA in revising the PPL remains unclear (13). It is within this backdrop that HTA and explicit health priority-setting approach is being institutionalized.

### Key institutions and actors in HTA production and use in Ethiopia

Several institutions and actors play critical roles in the generation and/or use of one or more components of HTA. Governmental agencies, like the Ministry of Health, Ethiopian Pharmaceuticals Supply Services, Ethiopian Health Insurance Service, and the Ethiopian Food and Drug Authority, hold substantial power and influence in the HTA and priority-setting process. The Ministry of Health, in particular, has a significant impact on HTA processes, involving tasks from defining and updating the essential health service package and prioritizing health services for exemption, to promoting cost-effective health technologies and addressing risks associated with new health technologies. Its dedicated Health Financing, Economics and Partnership Team supports and institutionalizes various evidence on health financing, including health expenditure tracking, efficient resource allocation, and conducting cost-effectiveness analysis and health technology assessments. The Ethiopian Food and Drug Administration is a government agency established with the goal of ensuring the safety and quality of health technologies and services. Two primary sectors of the Ethiopian Food and Drug Administration, the Medicines or Drug sector, and the Medical Devices sector, are involved in evaluating and regulating medicines and medical devices, aligning with the country’s health system demands, the Ministry of Health’s strategy, and international standards. Yet, they acknowledge a gap in their scope concerning value-for-money assessments. A participant from the Ethiopian Food and Drug Administration explained:

*“Our main focus is on medicine and medical devices, assessing them according to WHO standards for product registration and market authorisation. Every five years, we reassess existing health technologies to determine their continued use, modification, or exit from the system. However, we don’t evaluate costs and cost-effectiveness as this aspect is beyond our operational realm.” KII-3*

The Ethiopian Pharmaceuticals Supply Service is instrumental in procuring pharmaceuticals, medical equipment, and laboratory supplies. Its procurement process is guided by a Pharmaceutical List, which is prepared in response to health system demands and in a deliberative process involving various stakeholders. However, while Ethiopian Pharmaceuticals Supply Service’s Pharmaceuticals Supply Transformation Plan II (2020/21-2029/30) emphasizes evidence-informed decision-making, especially in designing and revising this procurement list, it does not specify how these evidence-informed practices are integrated into daily operations nor does it identify the responsible departments, highlighting a gap in operationalizing these principles effectively within the agency. The Ethiopian Health Insurance Service is another crucial government agency where HTA is increasingly utilized. This agency plays a leading role in coordinating and implementing both Community-Based Health Insurance and Social Health Insurance systems across Ethiopia. The use of HTA by the Ethiopian Health Insurance Service is growing, particularly in its strategic purchasing efforts. This includes the design of health insurance benefit packages and medicine lists, the implementation of various provider payment mechanisms, and the development of strategies, legal frameworks, and national standards.

Research institutions, such as the Armauer Hansen Research Institute, Ethiopian Public Health Institute, and several universities, are actively involved in research activities related to HTA and health priority setting. For instance, the Knowledge Translation Directorate of the Ethiopian Public Health Institute is actively engaged in synthesizing evidence and producing policy briefs and other knowledge products for policymakers. Similarly, Armauer Hansen Research Institute, despite not having a specialized HTA unit, undertakes HTA-related activities within its broader research framework, focusing on trials and studies to assess the efficacy, safety, and cost-effectiveness of health technologies. Academics and research institutions within universities often emphasize safety and ethics in the context of market access for research purposes. This focus, while important, tends to overlook crucial elements like cost-effectiveness and health system impacts.

*“In our research centers, collaboration on testing drugs, vaccines, and technologies not approved in their origin countries is frequent. These often enter Ethiopia without the Ethiopian Food and Drug Administration approval. Our HTA primarily evaluates ethical and safety concerns within research settings, as per our institution’s IRB guidelines. Unfortunately, this often leads to overlooking the financial aspects, value for money, and scalability of these technologies, despite our broader understanding of HTA as an all-encompassing concept.” - KII-1*

The existing HTA initiatives within these government agencies and research institutions are often fragmented and lack systematic coordination. There is also a lack of clarity and transparency in the criteria and methods for priority setting employed in designing and revising crucial policy documents, such as the Ethiopian Pharmaceutical Supply Agency’s Pharmaceuticals Procurement List and the Health Insurance Service’s Health Benefits Package. The effective implementation and routine application of HTA and explicit priority-setting approaches are further impeded by the absence of dedicated HTA units within these agencies, and the limited capacity specific to HTA, which is compounded by a rapid turnover of experts in this field. Furthermore, inadequate collaboration between institutions and weak linkages between research and policymaking exacerbate these challenges.

The involvement of other key stakeholders, such as professional associations, the private sector, patients, and the public, is notably lacking. Respondents from the Ethiopian Medical Laboratory Association and the Ethiopia Pharmaceutical Association highlight this gap. These associations, despite their ability to provide valuable technical and contextual expertise, find their influence in the HTA arena limited. This limitation often arises from weak, ongoing relationships with decision-makers and a lack of substantial recognition and involvement in policy-making processes. The minimal involvement from these stakeholders is further exacerbated by the inadequate mechanisms for patient/public participation, and lack of regulatory frameworks that set out transparent mechanisms for collaboration and manage and (potential) conflicts of interest. Table 2 summarizes the challenges and opportunities in various stakeholder groups in the context of HTA in Ethiopia.

**Table 2.** Challenges and opportunities in various stakeholder groups in the context of HTA in Ethiopia

Stakeholder group	Challenges	Opportunities
Government Agencies – general (incl Ministry of Health)	Lack of well institutionalized HTA process, Limited funding, Insufficient technical expertise	Strong influence on health policy, Control over health funding, Direct role in HTA process
Ethiopian Pharmaceuticals Supply Service	Limited HTA capacity, Limited HTA-related activities	Extensive supply chain, Access to a broad range of health technologies
Ethiopian Food and Drug Authority	Insufficient capacity for comprehensive HTA	Key role in health technology regulation, Access to safety and efficacy data
Ethiopian Health Insurance Services	Limited use of HTA in coverage and reimbursement decisions	Potential to use HTA for better decision-making, Access to data on health services utilization
Ethiopian Public Health Institute and Armauer Hansen Research Institute	Limited capacity and scope for HTA	Potential to develop HTA units, Strong research capabilities
Public Universities	Limited HTA training and research, Insufficient collaboration with health sector	Potential to develop HTA capacity through education and research, Access to academic resources
Professional associations	Limited involvement in HTA process, Insufficient capacity for evidence-informed advocacy	Potential to contribute to HTA process, Access to professional expertise
Patient groups and the public	Limited understanding and involvement in HTA, Inadequate mechanisms for public participation	Potential to contribute to HTA process through patient and public involvement, Access to patient experiences and preferences

### Inter-agency dynamics and interests among actors

Health priority-setting in Ethiopia involves a complex network of agencies and actors at national and local levels, each with distinct interests and perspectives on health sector development and HTA. Key agencies, including the Ethiopian Food and Drug Authority, Ethiopian Pharmaceuticals Supply Service, and Ethiopian Health Insurance Service, operate with a degree of autonomy while aligning with the Ministry of Health’s overarching strategies. The Food and Drug Authority focuses on assessing the safety, efficacy, and registration of health products; the Pharmaceuticals Supply Agency manages the procurement and supply of medicines and medical devices; and the Health Insurance Service oversees service delivery and coverage through health insurance schemes. However, their autonomy can sometimes result in misalignments, such as discrepancies between the Pharmaceuticals Supply Service’s prioritized medicines list and the Food and Drug Authority’s registered list. These challenges have been mitigated through established forums fostering continuous communication and role alignment. An official from the Food and Drug Authority highlighted their collaborative approach to resolving such issues:

*“Aligning Ethiopian Pharmaceuticals Supply Service’s supply priorities with our registration list posed significant challenges and led to misunderstandings. To mitigate this, we established a forum for ongoing communication with EPSI. This effort ensures our roles and focuses are not only aligned but also complementary. In instances where EPSI imports products that are unregistered but safe and in high demand, our proactive engagement with importers and manufacturers has been crucial for facilitating their registration.” – KII-4*

The institutionalization of HTA involves multiple agencies with overlapping mandates and legislative functions, which can create bureaucratic challenges and conflicts. These dynamics risk horizontal fragmentation, as agencies may compete for authority, budgets, personnel, and influence, undermining a unified approach to HTA implementation. To address these challenges, the TWG, comprising representatives from key government agencies and stakeholders, adopted a participatory approach in developing the national HTA institutionalization roadmap. Over a year, workshops were held to establish common ground, build trust, and enhance credibility among stakeholders. A centralized HTA agency was a recurring recommendation from key informants and a primary suggestion in the roadmap. Such an agency, supported by dedicated focal persons or working groups across relevant entities, was proposed to mitigate inter-agency conflicts and streamline HTA processes. This centralized approach is seen as a way to bring various government bodies together, streamlining HTA processes and fostering more effective collaboration that can ensure a more cohesive and effective HTA system.

### Donors and external actors

A key factor influencing the priority-setting process within the multi-donor funding context is the influence of external actors. In the early stages of operationalizing the HTA institutionalization roadmap, managing donor interests and navigating political dynamics will require careful attention. Stakeholders expressed concerns about the long-term sustainability of HTA activities, particularly when they rely heavily on external funding. Shifts in donor priorities or funding reductions could jeopardize the continuity and growth of HTA initiatives.

### Capacity and skill gaps in conducting HTA

Of 121 participants invited to the HTA capacity assessment survey, 65 completed it, yielding a response rate of 53.7 percent. The survey revealed varying familiarity levels with HTA concepts and methods. On a Likert scale of 1–5, the highest-rated need for HTA output was “informing the design of the basic health benefits package” (4.54), followed by “informing the design of health service delivery” (4.48), “producing clinical guidelines or disease management pathways” (4.37), “coverage or reimbursement of individual health technologies” (4.26), and “provider payment or pay-for-performance schemes” (4.18). Respondents highlighted the urgent need for HTA outputs across various health technologies, including vaccines, medicines, medical devices, and public health initiatives. The highest familiarity was reported for literature searching and systematic reviews of quantitative evidence, with 25 respondents (38.5 percent) indicating a high level of knowledge. Budget impact analysis and its integration with clinical and economic analyses were the least familiar topics, with 22 respondents (33.8 percent) reporting limited familiarity. Other topics fell within the average familiarity range of 3.05 to 3.83 (Table 3).

**Table 3.** Familiarity with HTA concepts and methods among survey participants (ranking 1 (not at all) to 5 (very familiar))

Question	Not at all or slightly familiar	Moderately familiar	Very familiar	Mean and SD (from 1 to 5)
The purpose, type, and scope of HTA	10 (15.4)	45 (69.2)	10 (15.4)	3.55 (1.09)
The range of technologies that can be the subject of HTA (pharmaceuticals, vaccines, medical devices, public health initiatives, etc.)	7 (10.8)	45 (69.2)	13 (20)	3.69 (1.01)
Literature searching methods and systematic reviews of quantitative evidence	4 (6.2)	36 (55.4)	25 (38.5)	4.11 (0.92)
The different types of clinical study design and when they are used, and the concepts of an evidence hierarchy and risk of bias.	7 (10.8)	40 (61.5)	18 (27.7)	3.83 (1.02)
The purpose of economic evaluation in an HTA and the linkages between clinical and economic analyses	15 (23.1)	36 (55.4)	14 (21.5)	3.46 (1.24)
The role of Quality of Life and Patient Reported Outcome Measures (PROMs)	12 (18.5)	37 (56.9)	16 (24.6)	3.58 (1.25)
The role of budget impact analysis in HTA and linkages with clinical and economic analyses	22 (33.8)	36 (55.4)	7 (10.8)	3.05 (1.28)
The applicability and generalizability of clinical evidence due to inter/national differences in clinical context	14 (21.5)	38 (58.5)	13 (20)	3.49 (1.21)
The concept of equity and the links between equity, benefit, and public financing	17 (26.2)	38 (58.5)	10 (15.4)	3.38 (1.15)

The survey on respondent level of comfort in HTA skills (See Figure in supplementary file) revealed that a large majority, over 80 percent, reported moderate to high confidence in areas such as systematic review and meta-analysis, economic evaluation of health interventions, and measuring the economic burden of disease, indicating a strong proficiency in these areas across different work affiliations. However, confidence dropped in areas like public and patient engagement and measuring patient preferences, with over 30 percent of participants from all stakeholder categories and work affiliations acknowledging limited or non-existent skills. In the open-ended questions, respondents pinpointed key challenges hindering the production of HTA evidence. These included a scarcity of dedicated human resources, limited knowledge of HTA methodologies, challenges with data availability, and budgetary constraints. These factors were recognized as significant barriers impacting the efficient and effective generation of HTA evidence.

### Proposed HTA capability framework for Ethiopia

Based on the identified gaps and potential horizontal fragmentation in the institutionalization of HTA within Ethiopia, we propose an HTA Capability Framework, detailed in the supplementary file. This framework is informed by comprehensive survey findings, open-ended responses, interviews, and an extensive review of relevant literature (16–19). It categorizes critical HTA skills into core components, such as understanding health system and clinical contexts, evidence synthesis, health economic evaluation, and budget impact analysis. Additionally, it includes cross-cutting themes such as consumer and stakeholder engagement, as well as legal, ethical, and social considerations. The framework establishes proficiency levels along a continuum from foundational to highly advanced, providing contextualized benchmarks for skill assessment. Survey results, which predominantly identified respondents as having foundational or intermediate-level skills, underscore the urgent need for targeted HTA training and capacity-building initiatives to strengthen Ethiopia’s HTA implementation efforts. This framework is designed to offer a structured approach for guiding capacity-building discussions and initiatives among stakeholders, including policymakers, academia, and development partners. It serves as a strategic tool for the Ministry of Health and other key agencies to institutionalize HTA by identifying skill gaps and prioritizing targeted capacity-building efforts. Additionally, it provides a roadmap to standardize HTA adoption and integrate it into Ethiopia’s health system, fostering evidence-informed decision-making and ensuring alignment with national health priorities.

## Discussion

In this study, we explored how Ethiopia’s priority setting is adapting to broader health financing reforms and the practical implications of various contexts on the development and implementation of HTA. Our research has highlighted that the interaction of various governmental agencies involved in healthcare priority setting, each with its own interests and mandates, leads to challenges such as overlapping responsibilities. This complexity underscores the need for strong collaboration between these agencies, research organizations, and other stakeholders—supported by a strong legal framework—to effectively institutionalize HTA.

The effective implementation of HTA and the credibility and acceptance of decisions made by HTA bodies depend not only on robust data but also on extensive and meaningful engagement with stakeholders (31;32) and the establishment of transparent frameworks that ensure decision-maker accountability (31;33). However, key stakeholders such as professional associations, the private sector, patients, and the general public remain underrepresented in Ethiopia’s health priority-setting processes. These groups, though not directly involved in HTA, can play a crucial role in raising awareness, building capacity, and advocating for evidence-informed policies (34). For example, professional associations like the Ethiopian Medical Association could contribute significantly to HTA awareness and capacity-building efforts. Similarly, engaging patient groups and the public is essential to incorporate perspectives on the real-world impact and societal acceptance of health technologies (34;35). However, their participation in Ethiopia’s HTA process is limited, often due to a lack of awareness and understanding of HTA, and the absence of formal mechanisms for their systematic inclusion in decision-making. Enhancing their engagement would not only increase the transparency and legitimacy of HTA but also ensure that decisions better reflect community needs and preferences.

Donors and international agencies play a significant role in Ethiopia’s health sector by providing financial resources and technical expertise, which influence priority-setting processes. The Ministry of Health centrally manages these funds through the “One-Plan, One-Budget, One-Report” system, redistributing them to Regional Health Bureaus and Woreda Health Offices. While this approach facilitates coordination, donor-driven priorities and protocols can shape health system objectives in ways that may not fully align with local needs. Stakeholders expressed concerns about the sustainability of HTA-related activities, particularly if they rely heavily on external funding, as shifts in donor priorities or funding reductions could disrupt progress. This raises critical questions about the ability to maintain and grow HTA activities independently, and highlights the importance of creating sustainable, locally led strategies for the implementation and integration of HTA into the national healthcare system. Similar trends of external actor influence in priority-setting processes have been observed in other LMICs operating within multi-donor funding contexts (36;37). Addressing these challenges requires balancing donor interests with local priorities and developing sustainable, locally led strategies to integrate HTA into Ethiopia’s healthcare system, ensuring its relevance, effectiveness, and long-term viability.

Our study also identified several challenges in HTA expertise in Ethiopia, including limited technical expertise, high turnover of HTA professionals, and gaps in specific areas of HTA knowledge. While several HTA capacity-building initiatives, including those led by development partners, are in place, they often overlook the organizational and institutional capacity, diminishing their long-term effectiveness. Survey respondents demonstrated moderate familiarity and skills with most HTA topics, yet areas such as budget impact analysis and measuring patient/public preferences were less understood. Considering that a significant majority of respondents (84.6 percent) were affiliated with research institutes or universities, it is important to note that the higher reported proficiency in HTA skills might potentially overestimate the overall expertise within the broader HTA community. Nonetheless, the findings highlight an imperative for tailored education and training programs aimed at enhancing understanding and expertise in vital HTA areas, applicable to both the academic sphere and health agencies.

Higher education institutions and government research bodies are critical in addressing these capacity gaps. Several universities, including Addis Ababa University, University of Gondar, and Jimma University, have incorporated HTA components into their health economics and policy courses and conduct HTA-related research in collaboration with international partners. Additionally, research undertaken by these universities, often in partnership with international collaborators, frequently addresses topics relevant to HTA. Strengthening collaborations among universities, government entities, and stakeholders is essential to create an environment where HTA becomes integral to health research and policy development, ultimately reinforcing Ethiopia’s healthcare system.

Ethiopia can draw valuable lessons from other LMICs, such as Kenya, Ghana, and South Africa, that have successfully institutionalized HTA (19;38). In Kenya, a government-led initiative established a dedicated HTA office and fostered multi-stakeholder engagement across government, academia, and the private sector, aligning HTA processes with national health policy goals (39). Similarly, Ghana integrated HTA principles into national health policies through governance structures supported by international collaborations and capacity-building programs (40). Both countries emphasize the importance of stakeholder buy-in and political will, elements that are equally critical for Ethiopia’s context (41). South Africa’s experience further highlights how legal frameworks and policy reforms can facilitate HTA adoption (24). By learning from these experiences and leveraging its ongoing health sector reforms, Ethiopia has the opportunity to create a robust and sustainable HTA system that aligns with its unique context and needs.

## Conclusion

As Ethiopia advances towards efficient resource utilization and UHC through strategic health purchasing, the strategic development and implementation of HTA will play a critical role. The process of institutionalizing HTA in Ethiopia is influenced by a variety of factors, including managing intricate inter-agency dynamics, establishing a robust legal framework for an inclusive and transparent HTA process, building local capacity, and securing sustainable, domestically aligned funding. Addressing these challenges is key to ensuring that HTA becomes an integral, effective tool in Ethiopia’s healthcare decision-making, thereby contributing significantly to the country’s journey towards UHC.

## Supporting information

Erku et al. supplementary material Erku et al. supplementary material

## Supplementary material

The supplementary material for this article can be found at http://doi.org/10.1017/S0266462325000170.
