# Supplementary material for: Towards institutionalizing HTA in Ethiopia: using a political economy analysis to explore stakeholder perspectives and assessing capacity needs
Source: Int J Technol Assess Health Care. 2025 Apr 3;41(1):e24. doi: 10.1017/S0266462325000170 (PMC12019762; doi:10.1017/S0266462325000170)

Supplementary Table 1. Proposed HTA Capability Framework

| **Sub-themes** | **Foundational** | **Intermediate** | **Adept** | **Advanced** | **Highly advanced** |
| --- | --- | --- | --- | --- | --- |
| **Health System and Clinical Contexts** | | | | | |
| Health Policy Context | Basic awareness of Ethiopia's healthcare system structure and government roles in managing public and private health sectors. | Understanding of health policy tools and the roles of various governmental committees. | Comprehensive knowledge of Ethiopian health policy development, of different actors in Ethiopia's health system. | Analytical skills in Ethiopian health policy. Expertise in assessing health technology funding / reimbursement and understanding the historical and current HTA processes in Ethiopia. | Advanced ability to influence HTA use at a national level and integrate it with broader policy objectives in Ethiopia. |
| HTA as a Policy Tool | Basic understanding of the purpose and scope of HTA within the Ethiopian context.; Awareness of HTA types in Ethiopia (e.g., full vs rapid HTA). | Capability to assess the suitability of technologies for Ethiopian HTA processes.; Understanding of HTA types and their policy applications. | Proficient in applying HTA to address specific policy issues. | Skilled in prioritizing HTA for technologies aligning with national health objectives.; Proficiency in selecting suitable HTA types for policy questions. | Expert in integrating HTA into broader health policy initiatives in Ethiopia.; Expertise in developing HTA methodologies for Ethiopia |
| Clinical Context for HTA | Broad understanding of clinical care across various specialties, including principles of clinical pathways. | Ability to critically evaluate clinical pathways/algorithm/guidelines developed within Ethiopian healthcare context. | Developing clinical pathways/algorithm/ guidelines based on information available in Ethiopia, considering alternative technology uses in practice. | Understanding policy implications of clinical algorithm / guideline choices. | Expertise in developing evidence-based clinical pathways / practice guidelines. |
| **Evidence synthesis and critical appraisal** | | | | | |
| Systematic reviews and meta-analysis in HTA | Awareness of the importance of systematic reviews in HTA and understanding the basic principles and steps involved in conducting systematic reviews and meta-analysis. | Ability to conduct literature searches and data extraction for systematic reviews. Basic knowledge of statistical methods used in meta-analysis. | Developing systematic reviews with a focus on health technologies relevant to Ethiopia. Competence in critically assessing the quality of studies included in systematic reviews. | Ability to adapt and apply systematic review findings to Ethiopian healthcare scenarios. Proficiency in advanced statistical techniques for meta-analysis. | Leading and overseeing systematic reviews and meta-analyses for HTA. Expertise in teaching or mentoring others in conducting systematic reviews. |
| Critical appraisal of clinical evidence | Awareness of different types of clinical studies and their relevance to HTA in Ethiopia. Basic understanding of the principles of critical appraisal and its significance in HTA. | Ability to assess the validity and reliability of clinical studies. Understanding common biases in clinical research and their implications for HTA in Ethiopia. | Ability to interpret and summarize critical appraisal findings for HTA committees. Competence in appraising a wide range of clinical evidence and discerning its applicability to the Ethiopian healthcare system. | Advanced skills in critically evaluating complex clinical studies, including randomized controlled trials and observational studies. Capacity to lead critical appraisal processes and provide guidance on the inclusion of evidence in HTA. | Recognized expertise in critical appraisal with contributions to developing national guidelines or policies in HTA. Mentorship or training others in advanced critical appraisal techniques. |
| **Health Economic Evaluation** | | | | | |
| Health economic concepts and evaluation in HTA | Grasping fundamental health economics terminology and the role of economic evaluations in Ethiopia. | Developing skills in interpreting and applying various types of health economic evaluations specific to Ethiopia. | Gaining proficiency in building basic health economic models and understanding uncertainty factors in the Ethiopian healthcare context. | Mastering complex economic modeling and defining scenarios for policy decisions within Ethiopia's healthcare framework. | Achieving expertise in integrating health economic evaluations into policy decision-making, along with advanced modeling and analysis techniques. |
| Health economic model structure and data input | Gaining basic awareness of health economic model structures and an understanding of primary cost and clinical data sources in Ethiopia. | Developing skills in handling diverse health cost data and applying clinical data within Ethiopian health economic models, along with familiarity with various model structures. | Gaining the ability to assess the appropriateness of different models for Ethiopian health scenarios and to apply diverse cost and clinical data effectively within these models. | Developing proficiency in appraising health economic models and designing detailed costing and clinical data studies specific to the Ethiopian context. | Achieving expertise in developing new methods for health economic modeling in Ethiopia, coupled with advanced skills in handling, analyzing, and validating complex cost and clinical data inputs. |
| QoL and Utilities in HTA | Basic understanding of what constitutes quality of life, including familiarity with basic PROMs used. | Gaining a deeper insight into the various QoL and utility measures applicable in the Ethiopian healthcare context and their relevance. | Acquiring skills in selecting and applying appropriate utility measures for specific health conditions and contexts within Ethiopia. | Developing proficiency in critically evaluating and interpreting studies that use preference elicitation methods to assess quality of life and utilities in Ethiopia. | Achieving expertise in designing, conducting, and analyzing studies focused on preferences in healthcare within the Ethiopian context. |
| **Budget Impact Analysis** | | | | | |
| Financial Analysis and Pricing | Gaining a foundational understanding of healthcare pricing concepts and the basics of projecting future healthcare costs specific to the Ethiopian context. | Gaining knowledge in resource allocation strategies within Ethiopian healthcare, including the role and methodology of budget impact analysis in HTA. | Acquiring awareness of the Ethiopian government's strategies for healthcare resource allocation and the ability to interpret healthcare utilization data in this context. | Developing expertise in both public and private healthcare pricing mechanisms in Ethiopia and understanding how health policy settings impact utilization estimates. | Acquiring the ability to formulate comprehensive policies for healthcare resource allocation, aiming to ensure equity and mitigate financial risks. |
| Conducting Budget Impact Analyses | Developing a basic understanding of budget impact models and their role in the Ethiopian healthcare context. | Gaining proficiency in assessing and ensuring the accuracy of budget impact models, tailored to Ethiopian healthcare scenarios. | Acquiring the skills to construct and use basic budget impact models, relevant to Ethiopian health economics and policy settings. | Developing advanced skills in more complex budget impact modeling, incorporating a range of variables and scenarios specific to Ethiopia. | Achieving expertise in simulation modeling techniques for analyzing budget impact, specifically adapted to the Ethiopian healthcare system's needs and challenges. |
| **Cross-cutting themes** | | | | | |
| Consumer Engagement | Understanding the specific needs, preferences, and challenges faced by Ethiopian healthcare consumers, particularly in relation to HTA. | Recognizing and pinpointing elements within HTA that are relevant and important to consumers, such as accessibility, affordability, and cultural appropriateness. | Developing the ability to effectively convey the results and implications of HTA studies to consumers in a clear, understandable, and relevant manner. | Mastering various techniques and tools to gather consumer feedback and perspectives and integrating this input into HTA processes and analyses. | Demonstrating expert knowledge in crafting and implementing policies that enhance and support consumer involvement in HTA at a governmental level. |
| Stakeholder Engagement | Understanding the range of stakeholders involved in HTA, including their roles and interests. | Developing communication skills and strategies to interact with government bodies involved in HTA | Demonstrating effective engagement techniques with non-governmental organizations, industry representatives, healthcare providers, and patient advocacy groups. | Mastering negotiation skills and advanced communication strategies to handle complex stakeholder interactions, conflict resolution, and consensus building in HTA. | Excelling in conveying HTA-related information and findings to high-level policymakers and effectively utilizing media platforms for broader outreach and impact. |
| Legal Aspects | Understanding the basic legal framework surrounding HTA, including general regulations and guidelines. | Recognizing specific legal issues and considerations pertinent to HTA practices in Ethiopia. | Grasping the implications and potential legal outcomes of employing various health technologies within the Ethiopian context. | Developing a deeper comprehension of Ethiopian laws and how they specifically impact HTA, including interpretation of legal texts and precedents. | Ability to actively participate in the creation or amendment of laws and regulations governing HTA in Ethiopia |
| Ethical Aspects | Basic comprehension of the fundamental principles of ethical conduct in research. | Becoming aware of the specific bioethical issues and dilemmas that can arise in the context of HTA, such as patient consent, privacy, and equity. | Developing a deeper understanding of the bioethical principles specifically relevant to HTA. | Gaining proficiency in integrating and applying ethical principles when conducting HTA reviews. | Demonstrating advanced expertise in evaluating ethical dimensions of HTA and contributing to the development of new ethical frameworks. |
| Equity and Social Aspects | Understanding basic principles of healthcare equity and the specific challenges in the Ethiopian context. | Identifying how healthcare technologies impact equity in Ethiopia, including accessibility and cultural appropriateness. | Understanding the influence of cultural norms on healthcare choices in Ethiopian communities. | Applying knowledge of social determinants of health in HTA to ensure broader social responsiveness. | Developing policies for equitable HTA practices, aligning with international standards and local needs. |

**Supplementary Figure 1**. Level of comfort with Core and Soft HTA skills


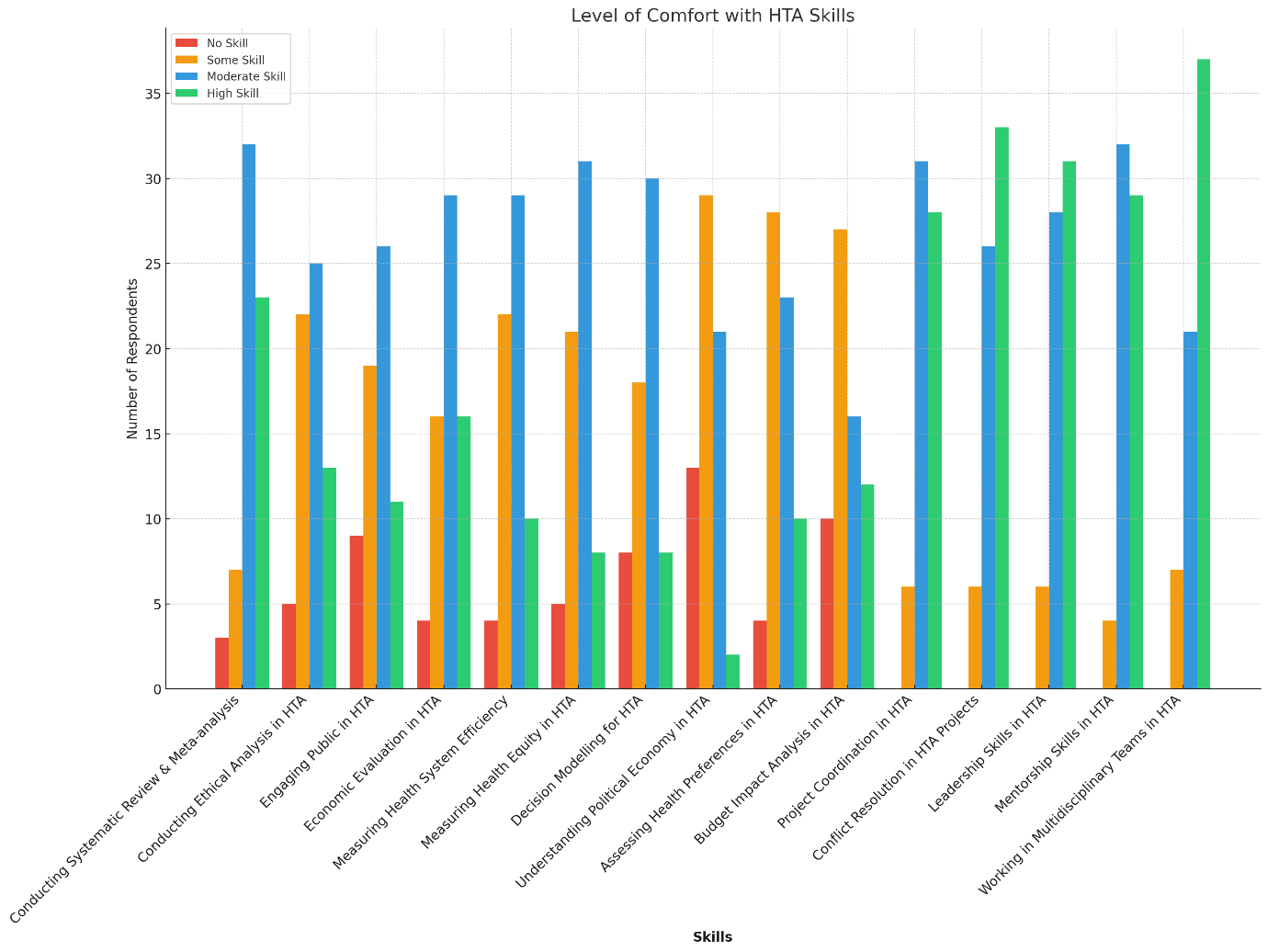

Supplement: Erku et al. supplementary material [file S0266462325000170sup001.docx]
